# The Influence of Residuals Combining Temperature and Reaction Time on Calcium Phosphate Transformation in a Precipitation Process

**DOI:** 10.3390/jfb13010009

**Published:** 2022-01-19

**Authors:** Farnaz Ghajeri, Klaus Leifer, Anders Larsson, Håkan Engqvist, Wei Xia

**Affiliations:** 1 Applied Material Science, Department of Engineering Science, Uppsala University, 75121 Uppsala, Sweden; farnaz.ghajeri@angstrom.uu.se (F.G.); Klaus.leifer@angstrom.uu.se (K.L.); hakan.engqvist@angstrom.uu.se (H.E.); 2 RISE Research Institutes of Sweden (RISE), 11428 Stockholm, Sweden; Anders.larsson@ri.se

**Keywords:** hydroxyapatite, precipitation, reaction temperature, reaction time, ions

## Abstract

Precipitation is one of the most common processes to synthesize hydroxyapatite, which is the human body’s mineral forming bone and teeth, and the golden bioceramic material for bone repair. Generally, the washing step is important in the precipitation method to remove the residuals in solution and to stabilize the phase transformation. However, the influence of residuals in combination with the reaction temperature and time, on calcium phosphate formation, is not well studied. This could help us with a better understanding of the typical synthesis process. We used a fixed starting ion concentration and pH in our study and did not adjust it during the reaction. XRD, FTIR, ICP-OES, and SEM have been used to analyze the samples. The results showed that combining residuals with both reaction temperature and time can significantly influence calcium phosphate formation and transformation. Dicalcium phosphate dihydrate formation and transformation are sensitive to temperature. Increasing temperature (60 °C) can inhibit the formation of acidic calcium phosphate or transform it to other phases, and further the particle size. It was also observed that high reaction temperature (60 °C) results in higher precipitation efficiency than room temperature. A low ion concentration combining reaction temperature and time could still significantly influence the calcium phosphate transformation during the drying.

## 1. Introduction

Bone regeneration is a complex system that involves many biological processes between materials, the environment, and substrate, and when all are in the suitable condition, a balance is achieved that leads to the desired results. Biomaterials are extensively used in this area, only if they are biologically stable, bio-compatible in the body, and cause no immune response [1]. Materials such as polymers, metals, and carbon-based ceramics have been used in clinical applications, but they are not efficient due to poor mechanical properties, low biocompatibility, and poor adhesion to human tissues [2]. Calcium phosphate (CaP)-based ceramics have been investigated as suitable biomaterials as they are found in human bone and have osteoconductive and osteoinductive characteristics which can help bone regeneration [3].

CaP-based ceramics are made of calcium cations (Ca^2+^) and phosphate anions (PO_4_^3−^, H_2_PO_4_^−^, and HPO_4_^2^), where different combinations of them in the reaction results in different types. For example, (Ca_x_ H_y_ (PO_4_)_z_·n H_2_O), where n:3–4.5 and Ca/P: 1.0–2.2, is an amorphous calcium phosphate according to Dorozhkin V.S. [4]. A lower Ca/P ratio is expected in calcium phosphates containing HPO_4_^2−^ ions instead of PO_4_^3−^. The mentioned amorphous phase can convert to crystalline phases depending on the composition of the reacting solution. For Ca/P ratios as low as 1.15, the material is unstable and converts to dicalcium phosphate dihydrate (DCPD) (CaHPO_4_·2H_2_O) and Ca/P ratio of 1.67 corresponds to hydroxyapatite (HA) (Ca_10_(PO_4_)_6_(OH)_2_) [5].

The conversion from ACP to apatite in a solution has been investigated [6,7,8,9] and the effect of pH, temperature, and the presence of foreign ions is considered as varying factors. According to an investigation by Boskey and Posner [7], carried out on different pH values (pH: 6.80–10.00), the conversion rate is considerably slower (5 h) at alkaline conditions (pH = 9.00). In more acidic solutions, the conversion rate becomes faster; however, there are some exceptions related to different sample preparation conditions or starting materials.

The conversion time for neutral solutions and 25 °C is reported to be 0.3 h [6]. At lower pH values (3.00–4.00), the conversion is much faster and DCPD is created in minutes [8]. The temperature has also effects on the conversion of ACP [7]. It is reported that the crystallization time decreases from 3 days to 20 min with increasing the temperature respectively from 10 °C to 37 °C.

Both the pH and Ca/P concentration of the solution has effects on the precipitation efficiency. According to Mekmene et al. [10], adjusting the pH to a constant value during the reaction increases the precipitation efficiency, and the initial Ca/P: 1.50 leads to the highest efficiency. Otherwise, in lower and higher Ca/P ratios, either the calcium or phosphate concentration is the limiting factor.

The drying method can also affect such materials. Freeze-drying [11] and air-drying [12] are some of the common drying methods. According to Vecstaudza et al., carbonated ACP is obtained when dried in air at 80 °C [13]. They have evaluated how drying methods and the pH value during synthesis impact the crystallization of carbonated ACP [14]. The result revealed that varying pH values during synthesis lead to differences in crystallization. They have also confirmed that regardless of pH value and drying method, ACP transforms into crystalline phases depending on the calcination at high temperature.

One issue always considered in a solution-based synthesis of CaP is the washing step before drying. The aim is to remove the effect of residual ions from the reaction solution, for example, to prevent the formation of by-products such as ammonium nitrate [15,16,17] if ammonium phosphate was used for the synthesis.

According to Dorozhkin et al., when the unreacted ions of Ca and P are removed from the reaction mixture during the washing step, the Ca/P molar ratio changes and it causes difficulties in the reproduction of every batch. Therefore, they have investigated and reported that the washing step could be avoided when producing HA and tricalcium phosphates (TCP) in temperatures below 100 °C where sintered at 1000 °C [18]. On the other hand, Sinusaite et.al. reported that the washing step can be used in controlling the production of desired TCP polymorph [19].

The common knowledge is that the residual ions would affect the crystal transformation and growth [19]. However, what will happen if we do not remove the residual ions and how the residual ions affect the crystals during drying is not clear. In this investigation, we have focused on the washing step to observe the challenges and their effect on the production of different CaP phases. The investigation can help us understand (1) if we can avoid the washing step to produce certain phases, and (2) if we can use less water or even ethanol for washing which is good for the environment and fits the Sustainable Development Goals (SDGs) [20]. This could help us understand the significance of the residual ions’ effect in a solution-based process.

## 2. Materials and Methods

CaCl_2_ from Acros Organics BVBA (Geel, Belgium) and Na_2_HPO_4_ from Sigma-Aldrich Sweden AB (Stockholm, Sweden) and deionized (DI) water were used in this experiment. A commonly used precipitation method was studied in this experiment [17,21,22,23,24,25], in which the effect of residual ions from the reaction solution on the calcium phosphate transformation combining the reaction temperature and time was analyzed.

In this method, precipitation was initiated by rapid mixing of 0.02 mol/L CaCl_2_ in 0.5 L H_2_O (sol.1) and 0.02 mol/L Na_2_HPO_4_ in 0.5 L H_2_O (sol.2). The solutions were prepared with deionized water and the total concentration of the used reagents in H_2_O was 20 mmol/L.

The pH of each solution was analyzed before and after mixing. Experiments were performed at room temperature (RT ≈ 21 °C) and 60 °C. Two processes for purifying the materials after reaction were chosen. (1) Residual ions were removed from the precipitate by twice filtering using a membrane with 0.4 µm pore size and washing with deionized water (DI) water and ethanol; this process is labeled as W in samples. (2) By centrifuging with 5000 rpm for 5 min, water was separated from the precipitate, whereas the residual ions were remaining in the precipitate; this process is labeled as NotW in samples.

The resulting paste samples were mixed with ethanol to stop the reaction and stored at RT before the analysis. The reaction time was varied from 1 min (min) to 1 h (h). Table 1 illustrates the samples made with the four different processes. The samples are labeled as reaction temperature/W or NotW/reaction time, for example, a sample made at RT and washed through the process (1) with 1 min reaction time is labelled as 21 °C/W/1 and a sample made at 60 °C that is centrifuged according to process (2) with 30 min reaction time is labelled as 60 °C/NotW/30.

The phases of the dried samples were analyzed using a Bruker D8 Twin-Twin powder X-ray diffractometer (XRD). To prepare the samples for XRD analysis, the samples that were stored in ethanol, ultrasound treated for 1 min, one drop of the resulting solution was added to the sample holder (silicon wafer), and dried under the heating lamp. In this study, we tried to keep the synthesis process the same, including drying method and time. Therefore, we could compare the relevant results. The XRD scan was done in the angular range of 2 θ = 7−60°. The XRD results were analyzed using High Score plus and Profex software.

Changes in the morphology of the precipitates were observed via Zeiss Merlin Scanning Electron Microscopy (SEM) from Germany. The images were obtained at 5 and 10 kV acceleration voltages (SE2, magnifications up to 500 k). To prepare the samples for SEM analysis, the sample was diluted in ethanol, ultrasound treated for 1 min, and one drop was dried on the SEM sample holder (made of aluminium); the sample was then gold/palladium coated to decrease the charging effect. The SEM results are analyzed using Gatan Digital Micrograph.

Fourier Transform Infrared Spectroscopy (FTIR) investigation was carried out using a Bruker Tensor 27 spectrometer. The analysis was performed as the following: a background analysis has been conducted with no sample under the measurement tip and the experiment followed with placing a small amount of powder sample under the FTIR measurement tip and running the measurement. An FTIR graph was then provided by the instrument software. The spectra were recorded in the range of 400−4000 cm^−1^.

Elemental analysis was performed using Inductively coupled plasma optical emission spectroscopy (ICP-OES) from Perkin Elmer, Avio 200. To analyze samples under ICP, a blank solution (2% HNO_3_) was used as a baseline for the measurements. Calibration was performed with stock standard solutions from PerkinElmer. Diluted samples were dissolved either in 2% HNO_3_ or ASTM water (depending on the standard solutions base) and filtered prior to analysis to avoid contamination and saturation of the instrument.

## 3. Results

The samples that were prepared as in Section 2 were analyzed under different analysis methods and the results are as below:

### 3.1. XRD Analysis

**Figure 1:**
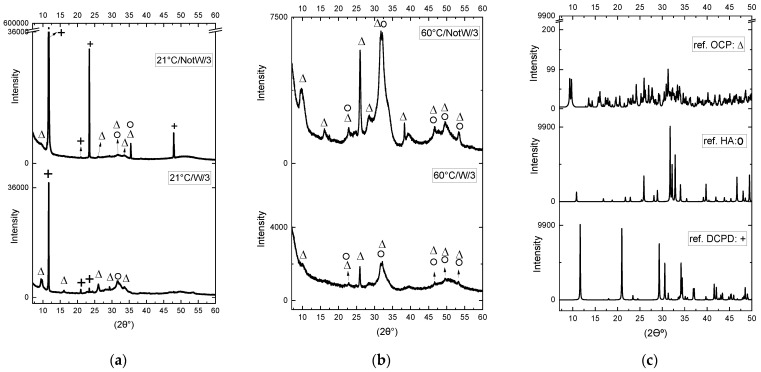
XRD analysis results for (**a**) process 1 (21 °C/W/3) and 2 (21 °C/NotW/3) (**b**) process 3 (60 °C/W/3) and 4 (60 °C/NotW/3) obtained after 3 min reaction and (**c**) the reference powder patterns from Inorganic Crystal Structure Database (ICSD) for DCPD (PDF no: 00-009-0077), HA (PDF no: 00-064-0738) and OCP (PDF no: 04-013-3883) that are provided for comparison. The peaks in (**a**,**b**) are marked as Δ, + and O which matches respectively with the peaks at the ref. OCP, ref. DCPD and ref. HA/ nanocrystalline apatite/. Some peaks overlap in both OCP and HA. The high intensity in the starting point of the graph in 2 θ: 7° is due to the device not being suitable for low angle measurements and considered as error.

**Figure 2:**
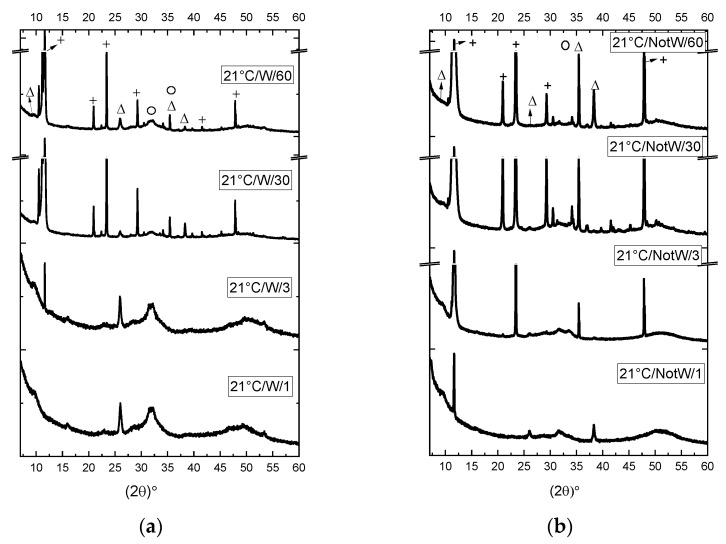

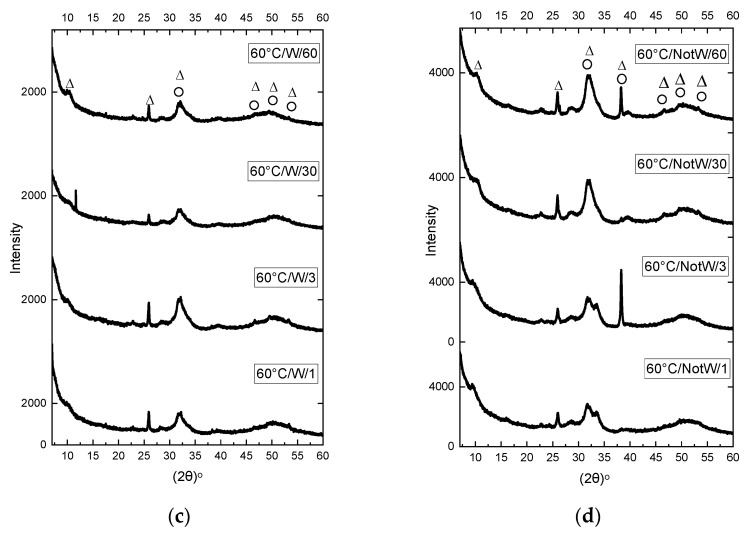
XRD results of (**a**) process 1 (21 °C/W) (**b**) process 2 (21 °C/NotW) (**c**) Process 3 (60 °C /W) (**d**) process 4 (60 °C/NotW) with reaction time 1 min to 1 h. The peaks are marked as Δ, +, and O which matches respectively with peaks at the ref. OCP, ref. DCPD and ref. HA/nanocrystalline apatite. Some peaks overlap in both OCP and HA. There is a break in the Y-axis of the graph in a and b due to the high intensity of the peak at 11.6 that otherwise hinders the observation of the other peaks with lower intensity.

The XRD patterns of the specimens, prepared according to the four processes in Table 1, are shown in Figure 1 and Figure 2. Figure 1 shows the XRD patterns obtained after 3 min for the 4 processes. Figure 2 shows the time influence on the four processes.

When comparing the XRD patterns, we compared the peak points in XRD patterns of the samples (Figure 1a,b) to the reference peaks in Figure 1c and confirmed if the peak points match the reference peaks. When identifying the peaks, we focused on the phases, not the intensity or width as the intensity and width of the peak in an XRD pattern is related to crystal orientation and crystal morphology, which can get affected by sample preparation, calibration errors, etc. [26]. It also has to do with the nanocrystalline nature of the sample.

The dominating peak in the XRD pattern of the samples made at RT, as shown in Figure 1a, is a peak at 2 θ: 11.6°, which matches the DCPD pattern, shown in Figure 1c. When comparing the W and NotW samples with 3 min reaction, respectively (21 °C/W/3) and (21 °C/NotW/3) in Figure 1a, the DCPD peaks are stronger in NotW samples, indicating that the grains grow much faster in NotW samples. The dominating peak in the XRD pattern of samples made at 60 °C, as shown in Figure 1b, is a broad peak between 30–35 that matches nanocrystalline apatite as reported in [27], indicating the nanocrystalline nature of the grains. However, there are some peaks on the same area that matches with HA and OCP patterns (see HA and OCP in Figure 1c). Comparing the XRD patterns in Figure 1a,b, the peak observed in 2 θ = 11.6° in Figure 1a is not observed in Figure 1b. That peak belongs to DCPD, which indicates that DCPD is produced in samples made at room temperature, but not in samples made at 60 °C.

When comparing W and NotW samples made at 60 °C with 3 min reaction, respectively (60 °C/W/3) and (60 °C/NotW/3) in Figure 1b, the grain growth is faster in the NotW sample; however, the difference is not as significant as in RT samples (Figure 1a). This indicates that the washing condition has a major influence on samples made in RT and a minor influence on samples made at 60 °C.

In a 60 °C reaction, ions are consumed within the first minute of the reaction and small crystals form, and these small crystals will create bigger clusters by the time, but the crystal growth is stopped. Increased temperature will decrease the solubility of hydroxyapatite and make it stable. An OCP pattern is detected in all samples (see Figure 1a,b and reference OCP pattern in Figure 1c). The broad shape of the peaks in around 30 and 50, and matches the nanocrystalline apatite and ACP pattern reported by [27], indicating that it is partly amorphous.

According to Figure 2a, 21°C/W/1–60, the dominating peak in the sample with 1 min reaction, is a broad peak around 30–35, that peak together with 3 other broad peaks between 45–55, matches the pattern reported for nanocrystalline apatite [27]. The mentioned peak exists even after 60 min; however, DCPD is created in the sample with 3 min reaction and is the dominating peak for samples with 3 min and longer reaction time. This shows that nanocrystalline apatite is created at the beginning of the reaction and stays in the sample even after 60 min; however, it is not the dominating peak even 3 min after the reaction.

Comparing the W and NotW samples made at RT with 1 min reaction, respectively 21 °C/W/1 in Figure 2a and 21°/NotW/1 in Figure 2b, the mentioned peak at 11.6 that matches ref. DCPD in Figure 2c is observed in the NotW sample; however, it is not observed in the W sample. This may have a relation to the existence of residual ions in NotW samples; however, it can also be due to the washing step if the crystal size of DCPD is below the pore size of the filter 0.4 µm in 1 min reaction time. Observing the samples made at 60 °C, as shown in Figure 2c,d, OCP and nanocrystalline apatite co-exist in the XRD patterns. OCP with two peaks in 9.6° and 9.8° is the dominating peak for W samples, as shown in Figure 2c, and it does not have a significant change even after a 60 min reaction. In NotW samples, as shown in Figure 2d, the broad peak for nanocrystalline apatite is getting stronger with a longer reaction time.

### 3.2. pH Analysis

**Figure 3:**
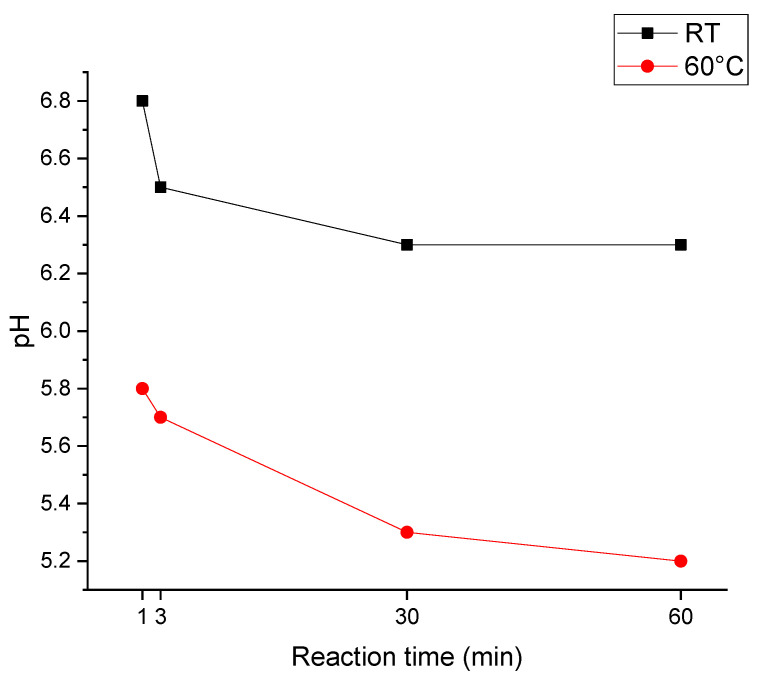
pH value of the samples measured during the reaction.

The pH value is one of the important factors in the synthesis of calcium phosphate as the varying pH value during synthesis can lead to differences in crystallization [28]. In this investigation, the pH value was measured during the reaction to relate its variation to its potential impact on the resulting structure. It has not been adjusted to any predefined value; we rather let the pH drift during the reaction and observed how it impacts the structure. The pH value for every sample was measured in solutions before and after mixing, and the results are as in Figure 3 and Table 2.

As can be seen in Table 2 and Figure 3, the pH value of the starting solution differs and depends on the reaction temperature. It decreases gradually after mixing the two solutions from the first minute to 60 min. The pH value of the sample made at room temperature was measured: 5.90, after seven days.

We observed that the pH value for the synthesis at 60 °C decreased by 0.6, whereas the pH value for the synthesis at RT decreased by about 0.4 in the first 60 min of the reaction. This indicates that in the case of the synthesis at 60 °C, reactions are favored where more protons are emitted into the solution during the reaction.

As shown in the XRD analysis, the dominant reaction products in this synthesis are DCPD for the RT sample and OCP and HA/nanocrystalline apatite for the 60 °C samples. Starting from the initial stoichiometry of the initial solution, the reactions, leading to these three salts, are as follows [29,30]:

Ca^2+^ + HPO_4_^2−^ → CaHPO_4_ (**DCPD**) (1)

10 CaHPO_4_ + 2H_2_O → Ca_10_(PO_4_)_6_ (OH)_2_ (**HA**) + 4H_3_PO_4_ (2)

8Ca ^2+^ + 8(HPO_4_)^2−^ → Ca_8_ H_2_(PO_4_)_6_ (**OCP**) + 2 H_3_PO_4_ 5Ca_8_ H_2_(PO_4_)_6_ + 8 H_2_O → 4Ca_10_ (PO_4_)_6_ (OH)_2_ (**HA**) + 6 H_3_PO_4_ (3)

According to the above Formula (1) and (2), it is observed that the synthesis of OCP and HA produces an increased acidity of the solution. Other groups have reported that OCP and HA can be transformed from DCPD [31,32] in acidic conditions. In our study, we observed DCPD peak in XRD results of our room temperature samples in Figure 2a,b; it may happen that in samples made at 60 °C, the DCPD is transformed to HA and OCP, and therefore is not present in XRD results (see Figure 2c,d). Therefore, the observation of more strongly drifting pH towards higher acidity of the 60 degrees C sample is in good agreement with the observation of the dominating fabrication of OCP and HA.

### 3.3. FTIR Analysis

**Figure 4:**
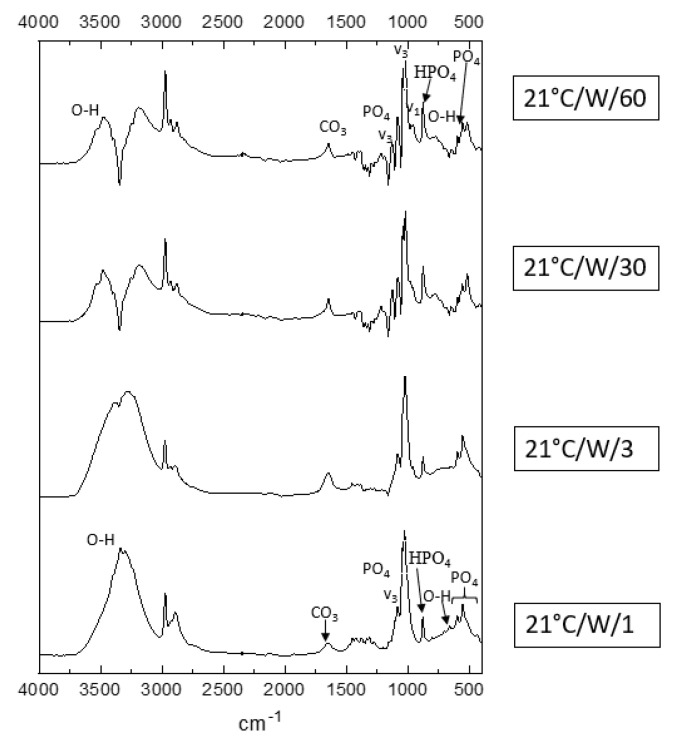
FTIR results for samples from process 1 (21 °C/W/1–60).

Figure 4 illustrates the FTIR results of samples from process 1, 21 °C/W with varying reaction times from 1 min to 60 min.

Comparing the spectra in Figure 4 to the FTIR spectra of DCPD, HA, OCP, and nanocrystalline apatite reported in [27], in the region (500–1200 cm^−1^), the spectrum matches OCP and nanocrystalline apatite (with a peak at 877 cm^−1^ and two peaks at 500–550 cm^−1^) in 21 °C/W/1 and brushite is seen on 21 °C/W/3, 21 °C/W/30, and 21 °C/W/60. This result confirms the XRD results.

Considering the sample 21 °C/W/1 in Figure 3, the peaks around 2900–3000 cm^−1^ are typically C-H modes and probably originate from residual ethanol in the sample also the noise near 3300 cm^−1^ and 1500 to 1000 cm^−1^ can result from the absorption of water vapor (H_2_O) or ethanol, indicating that the sample was not fully dried before the analysis [33,34,35].

Over time, there is a dip observed in the water region (3300 cm^−1^) that is one particular OH stretching mode, indicating that the sample started to lose some water or some of the ethanol gets lost (samples prepared in ethanol before drying; see Section 2). According to a study [36], bulk water has a specific OH stretching frequency around 3400 cm^−1^. There are multiple -OH environments observed in the sample [37]; however, only one of them is removed from the sample during the reaction time that created the mentioned dip which can correspond to the removal of bulk water as mentioned. Oliver et al. investigated the dehydration process by FTIR and reported the changes in the resulting spectra.

There are shifts of the bands on 3000–3400 cm^−1^ when comparing the 4 samples in Figure 4, according to the FTIR pattern for DCPD and OCP, reported by [27,38,39], the patterns in Figure 4 are similar to OCP in samples 21C/W/1 and 21C/W/3, whereas, in samples 21C/W/30 and 21C/W/60, the pattern is more similar to DCPD; this confirms the XRD results that DCPD gets created at 21 °C/W/3 and gets stronger in 21 °C/W/30 and 21 °C/W/60.

### 3.4. SEM Analysis

**Figure 5:**
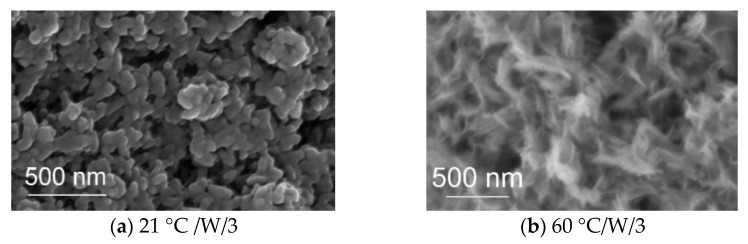
SEM images of the W samples (filter cleaned via process (1)) made at *(***a**) 21 °C and *(***b**) 60 °C for 3 min.

**Figure 6:**
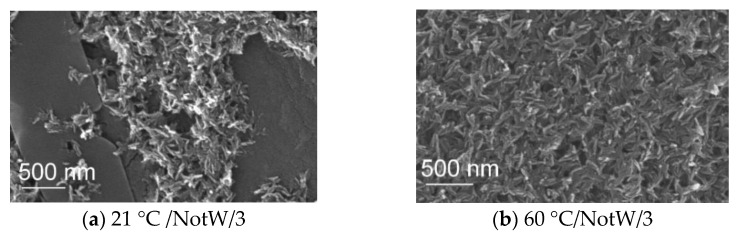
SEM image of the NotW samples (centrifuged via process 2), (**a**) 21 °C and (**b**) 60 °C with 3 min reaction.

The SEM analysis was conducted to observe the morphology and the particle size. Figure 5 illustrates two SEM images that belong to W samples synthesized at RT and 60 °C for a 3 min reaction. Figure 6 shows the NotW samples made at RT and 60 °C for 3 min reaction. It is observed that all samples contain aggregated nanoparticles. The shape of particles for the sample 21°C/W/3 (Figure 5a) is circular and other samples 60 °C /W/3, 21 °C /NotW/3, and 60 °C /NotW/3 (respectively Figure 5b and Figure 6a,b) contain plate-like and rod-like morphology.

**Figure 7:**
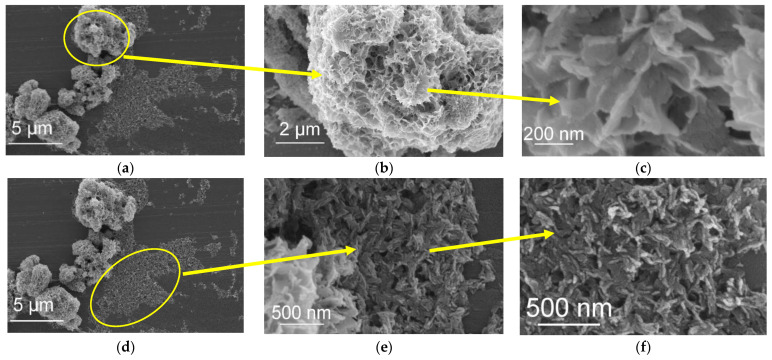
SEM image of the W sample (filter cleaned via process (1)) made at 21 °C with 1 min reaction (**a**) and (**d**) 21 °C/W/1. *(***b**) Magnified image of yellow marked part at Figure 7a. (**c**) Magnified image of Figure 7b. (**e**) Magnified image of the yellow marked part at Figure 7d. (**f**) Magnified image of Figure 7e.

Figure 7 shows the SEM image of sample 21°C/W/1. Clusters are observed in the SEM image, where the mentioned clusters have different shapes and sizes; there are larger (1–5 µm) clusters, yellow-marked at Figure 7a, made of smaller (100–200 nm) plate-like or flake-like morphology, as shown in Figure 7c, and there are smaller size clusters, yellow marked at Figure 7d that are made of shorter Nano-rod type (100 nm long) clusters, as illustrated in Figure 7f. There are also thickness differences in the mentioned parts.

**Figure 8:**
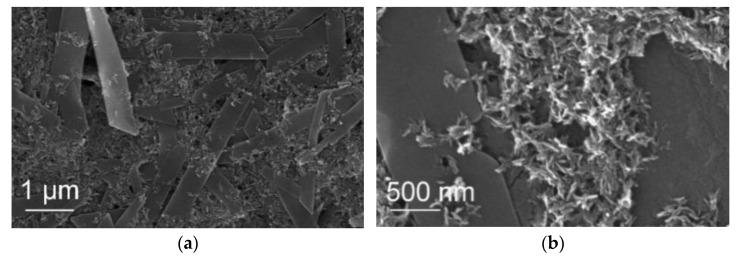
(**a**,**b**) SEM image of the NotW sample (centrifuged via process 2) made at 21°C with 3 min reaction (21 °C/NotW/3).

Figure 8 shows the SEM images of the sample 21 °C/NotW/3. It is observed that some flake-like crystals (approximately 1 µm wide) are created in 3 min that have not been observed in samples with a 1 min reaction. These crystals are observed together with smaller rod-like particles in the same area.

**Figure 9:**
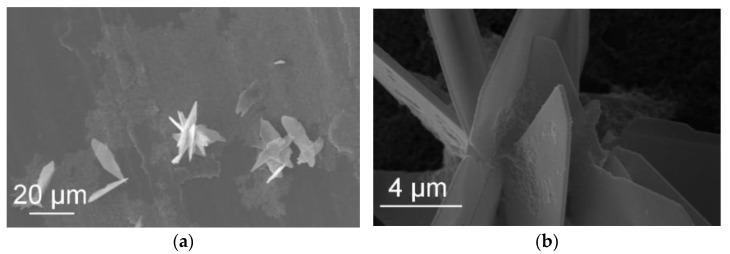
(**a**,**b**) SEM image of the NotW sample (centrifuged via process 2) made at 21 °C with 30 min reaction (21 °C/NotW/30).

Figure 9 illustrates the SEM images of the same sample after 30 min reaction (21 °C/NotW/30). The flake-like crystals seen in 3 min are much bigger in the sample with a 30 min reaction (approximately 20 µm wide and 50 µm long) where they created petal-like morphology that are made of flake-like particles. This structure has been observed for brushite in [40].

Considering the XRD results for the 21 °C/NotW sample in Figure 2b, the peak that is observed in 3 min and gets much stronger over the reaction time matches the DCPD peak. This indicates that the observed flake-like crystals are DCPD. Similar shape crystals are reported as DCPD in [41].

More SEM images with different magnification for samples made via the different synthesis conditions as in Table 1 are added as supplementary information in Supplementary Figures S1–S7.

### 3.5. Ca/P Ratios in Precipitates

**Figure 10:**
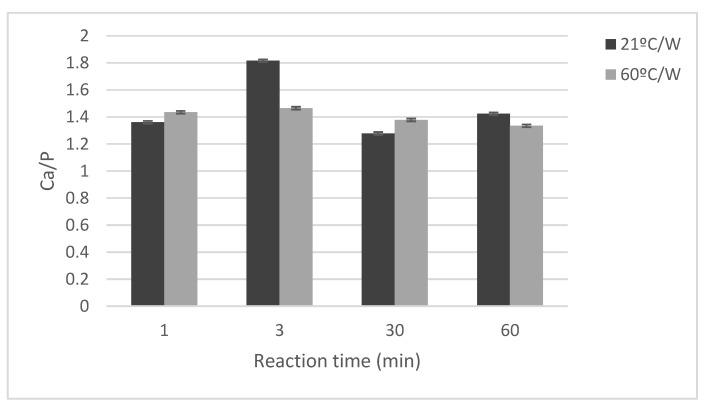
Total Ca/P molar ratio calculated according to ICP results. The black land red lines belong to dried samples made respectively at room temperature and 60 °C and washed via vacuum filtration. Every analysis is done three times and the average of each point is calculated and plotted in the graph below.

Figure 10 shows the changes in the Ca/P ratio of the particles prepared in the processes 1 (21 °C/W) and 3 (60 °C/W). For samples 60 °C/W, the Ca/P ratio has a slight increase between 1 and 3 min and a slow decrease until 60 min. However, for the sample (21 °C/W), the Ca/P ratio has changed significantly, it started around 1.35 (1 min), and increased to around 1.8 (3 min), and decreased to below 1.3 (30 min) and went back to around 1.45 (60 min). It might indicate an increase in precipitated calcium between 1 and 3 min or a decrease of phosphor and the opposite between 3 and 30 min. Whatever the reason, it is an indication of the transformation of the precipitate during this time.

**Figure 11:**
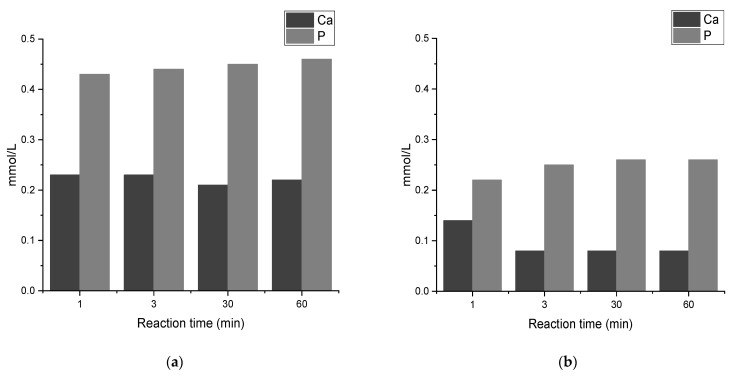
The amount of Ca and P in the rest solution that remains after centrifuge for sample made at (**a**) 21°C/NotW and (**b**) 60 °C/NotW.

Figure 11 shows the changes of Ca and P concentration existing as ions in the rest solution. The mentioned ions are separated from the sample by centrifugation and analyzed in the rest solution after centrifuge. For the sample prepared at RT, as shown in Figure 11a, the Ca ion concentration starts at 0.23 mmol/L (1 min and 3 min), it slightly decreases to 0.21 mmol/L in 30 min reaction, and reaches 0.22 mmol/L after 60 min reaction. On the other hand, the P ion concentration starts at 0.43 mmol/L (1 min), and it slightly increases to 0.44 mmol/L after 3 min, 0.45 mmol after 30 min, until it reaches 0.46 mmol/L after 60 min reaction. It is observed that there are more P ions than Ca ions in the rest solution.

Figure 11b shows the changes in concentration of Ca and P in the rest solution after centrifuge for samples made at 60 °C. The Ca ion concentration starts at 0.14 mmol/L (1 min) and it decreases to 0.8 mmol/L (at 3, 30, and 60 min). On the other hand, the P ion concentration starts at 0.22 mmol/L (1 min), and it slightly increases to 0.25 after 3 min and it reaches 0.26 mmol/L at 30 and 60 min. Comparing the ions in the rest solution of samples made at RT and 60 °C, as shown in Figure 11a,b, the number of ions is higher in RT samples than samples made at 60 °C, indicating that there are more Ca and P ions used in the precipitation reaction in samples made at 60 °C and therefore the precipitation efficiency is higher in this case.

## 4. Discussion

In this study, the typical precipitation process was used to observe the influence of the residual ions on the calcium phosphate formation together with the reaction time and temperature. We used CaCl_2_ and Na_2_HPO_4_ as the starting materials to remove the effect of ammonium nitrate by-products and focus on the washing step to observe the challenges and its effect on the production of different CaP phases. Na_2_HPO_4_ is a common chemical that has been used before, i.e., [17,21,22,23,24,25]. We have used a Ca:P ratio of 1:1, as a Ca:P between 1–2:1 is recommended to be efficient for bone health [42]. The starting pH was not adjusted before or during the reaction.

XRD analyses have shown the difference between the samples made at 21 °C and 60 °C and made via the two different purifying processes described in Section 2. The observed peaks at XRD results are compared to reference patterns, in Figure 1c, and the peaks that match the reference pattern are selected. For samples made at room temperature, as shown in Figure 1a and Figure 2a,b, patterns match phases of DCPD, OCP, and HA; however, DCPD with a strong peak at 2 θ: 11.6° is considered as the dominating phase. For samples made at 60 °C, as shown in Figure 1b and Figure 2c,d, the two phases of HA and OCP are identified as existing phases.

During the reaction, OCP and HA/nanocrystalline apatite phases coexist from the first minute. OCP has an identifying peak at 2 θ ≈ 5°, the existence of this peak is confirmed at 2 θ = 4.7° (conducted by XRD using a special program for low angle measurement) as well as other common OCP peaks. (See Supplementary Figure S8 in the Supplementary Material).

Comparing the W and NotW samples, filter cleaned and centrifuged respectively (see cleaning processed in Section 2), the NotW samples contain stronger peaks than W samples, indicating that the reaction is much faster in NotW samples.

We have observed that time has effects on the reaction of samples made at room temperature as the longer the time is the XRD peaks are stronger, indicating the crystallization matures over time. This confirms that both temperature and pH need to be fine-tuned for the crystallization to happen. FTIR results confirm the XRD results. On the other hand, at high temperature (60 °C) samples, the time does not influence the resulting phases, when comparing 1 min, 3 min, 30 min, and 60 min reaction times, indicating that the reaction is mature enough even after a 1 min reaction.

According to the pH analysis results (Figure 3), all samples are made in acidic media, which indicates the possible creation of DCPD as reported by Drouet et al. [27]. However, in this investigation, we did not observe DCPD at 60 °C, regardless of reaction time, even if the pH of the media is suitable for the precipitation of DCPD. According to an investigation made by Komlev et al., increasing the reaction temperature from 37 to 90 °C at a constant pH between 5.00–6.00, the phase composition varies from DCPD to a mixture of OCP and DCPD [43], where at 55 °C and pH: 6.00, the OCP (wt.) is 70%. Considering this information indicates the possibility of the phase change from DCPD to OCP in our experiment made at 60 °C and pH: 5.80–5.20. The formation of OCP from DCPD in acidic conditions (pH:6) is also reported in other investigations [31,32].

The crystal growth in the classical crystallization pathway is based on the idea that crystals in solution exist as discrete “particles” and are well separated from each other in solution. According to Ivanov et al., the growth occurs by mass transport of individual atoms or ions through the solution to the crystal interface [44]. However, more intricate mechanisms of nucleation can occur, namely, by oriented attachment of particles. According to this mechanism, primary nanoparticles are formed which aggregate, are ordered, and merge together giving coarser single-crystalline particles. The crystal growth occurs via the transport of colloidal particles rather than homogeneous solutions, i.e., Ostwald ripening mechanism [45]. The crystal growth based on colloids is a non-classical growth mechanism in contrast to the classical growth mechanism described by Ostwald ripening. According to the SEM images, in RT samples, when the reaction time changes from 1 min to 30 min, the particles are observed in the following stages:

1 min: aggregated clusters of three different shapes and sizes are observed as below:
  - Loose clusters 1–5 µm, made of spherical particles of 10 nm that are made of smaller particles.
  - Dens clusters made of Nano-rods (100 nm × 20 nm) similar to OCP [46].
  - Clusters 1–5 µm, made of flake-like morphology (200 × 100 nm)
3 min: two types of aggregates are observed as below:
  - Dens clusters made of Nano-rods (100 nm × 20 nm) similar to OCP [46].
  - Flake-like morphology of 5 × 0.5–1 µm similar to OCP [46] and DCPD [40].
30 min: two types of aggregates are observed as below:
  - Dens clusters made of Nano-rods (100 nm × 20 nm), similar to OCP [46].
  - Petal-like morphology that is made of flake-like particles of (25 µm × 5µm), similar to DCPD.

In 60 °C samples, when the reaction time changes from 1min to 1 h, the particles are observed in the following stages:

1 min: Dens clusters made of Nano-rods (100 nm × 20 nm), similar to OCP [46].
3 min: Dens clusters made of Nano-rods (100 nm × 20 nm), similar to OCP [46].
30 min: Dens clusters made of Nano-rods (100 nm × 20 nm), similar to OCP [46].

The observed nano-rod type particles are similar to that of OCP [46] and flake-like particles have been observed for brushite in [40]. However, the SEM results did not give enough information to follow the crystallization pathway and reveal the morphology and phase information at the nanoscale. We will therefore investigate the crystallization pathway using TEM in our next study. (See SEM images in Figure 5, Figure 6, Figure 7, Figure 8 and Figure 9 in Section 3.4 and Supplementary Figures S1–S7 provided as Supplementary Material).

The total Ca/P molar ratio of the samples is reported in Figure 10. It is observed that the Ca/P ratio varies during the reaction from 1 min to 60 min, which indicates that the phases observed in XRD results, especially in lower reaction times, are not stable. This study was designed to identify how removing/not-removing salts affected changes in phase at discrete points during the precipitation reaction. Therefore, phase analysis will be limited in accuracy, by the inherent variability of the ongoing chemical processes, and the method used to halt the reaction. Given the large variability, we had not included Rietveld’s analysis. The formation of phases, such as Hydroxyapatite, is not complete (not 100% crystalline, as determined by peak broadness and presence of semi-crystalline or amorphous peaks); therefore, the Rietveld refinements are expected to be limited in accuracy [47,48].

Considering the concentration of Ca and P ions in the rest solution after centrifuge, as shown in Figure 11a,b, it is observed that the amount of P ions is much higher than Ca ions, which indicates that more Ca ions have been used in the precipitation than P ions. This is due to the starting Ca/P: 1; it has been reported that in precipitations where starting Ca/P ratio is 1, the number of calcium limits the efficiency of precipitation [10]. When comparing the concentrations of Ca and P ions in the rest solution for samples made at RT and 60 °C, the concentrations of Ca and P ions are higher at RT than 60 °C, indicating that the efficiency of precipitation increases at 60 °C.

It was observed from XRD results that the existence of unreacted ions does not influence the samples made at 60 °C during the different reaction times; however, in samples made at room temperature, the peaks were observed to have higher intensity and more noise. Based on our observation, it is difficult to say if the low number of residual ions by themselves could have a significant influence, but combining with reaction temperature and time did.

## 5. Conclusions

The influence of residual ions on the calcium phosphate formation during a typical precipitation process has been investigated in aqueous solutions, varying reaction time and temperature, RT and 60 °C. We observed the influence of reaction temperature and time on the phase transformations. The reaction under room temperature is more sensitive to the reaction parameters and residuals in the solution as the formation of DCPD and its transformation are observed. High reaction temperature (60 °C) induces the formation of OCP and HA, and makes the particle size smaller than the ones obtained at room temperature. The SEM images did not show a significant difference between W and NotW samples (respectively filter cleaned via process 1 and centrifuged via process 2). Comparing the Ca and P ion concentrations in the rest solution remaining after centrifuge, it was observed that higher temperature resulted in less residual ions in the rest solution indicating the higher precipitation efficiency. The above results indicate that, in combination with reaction temperature and time, the residual ions can influence the formation of the intermediate phase, OCP and DCPD, and particle size when the starting ion concentrations are fixed.

## Figures and Tables

**Table 1:** Samples synthesis conditions.

	Temperatures		Cleaning		Reaction Time			
Process No.	RT	60 °C	W ^1^	Not-W ^2^	1 min	3 min	30 min	1 h
1	√		√		√	√	√	√
2	√			√	√	√	√	√
3		√	√		√	√	√	√
4		√		√	√	√	√	√

^1^ Cleaned according to process (1). ^2^ Cleaned according to process (2).

**Table 2:** Initial pH values measured before mixing the two solutions.

	Temperature		pH	
Process	RT	60 °C	pH sol.1 ^1^	pH sol.2 ^2^
1, 2	√		9.00	9.40
3, 4		√	6.00	8.70

^1^ Sol. 1: 0.02 mol/L CaCl_2_ in H_2_O. ^2^ Sol. 2: 0.02 mol/L Na_2_HPO_4_ in H_2_O.

## Data Availability

The data supporting reported results in this study are available upon request from the corresponding author.

## Supplementary Materials

The following are available online at https://www.mdpi.com/article/10.3390/jfb13010009/s1, Figure S1. SEM images of the sample made at 21 °C /W/1. Figure S2. (a–e) SEM images of the sample made at 21 °C /W/3. Figure S3. SEM images of the sample made at (a) 21 °C /W/30 and (b) 21 °C /W/60. Figure S4. (a–d) SEM images of samples made at 21 °C /NotW/1. Figure S5. SEM images of (a,b) samples made at 60 °C /NotW/1. Figure S6. SEM images of (a,b) samples made at 60 °C /NotW/3. Figure S7. SEM images of (a,b) samples made at 60 °C /NotW/30. Figure S8. XRD analysis conducted to observe the existence of peaks in low angels of 2 θ.

## References

[1] Hulbert S., Hench L., Forbers D., Bowman L. (1982). History of bioceramics. Ceram. Int..

[2] Hench L.L. (1991). Bioceramics: From Concept to Clinic. J. Am. Ceram. Soc..

[3] Jiwoon J., Kim J.H., Shim J.H., Hwang N.S., Heo C.Y. (2019). Bioactive calcium phosphate materials and applications in bone regeneration. Biomater. Res..

[4] Dorozhkin S.V. (2010). Amorphous calcium (ortho)phosphates. Acta Biomater..

[5] Ramesh S., Tan C.Y., Hamdi M., Sopyan I., Teng W.D. (2007). The influence of Ca/P ratio on the properties of hydroxyapatite bioceramics. Proceedings of the International Conference on Smart Materials and Nanotechnology in Engineering.

[6] Eanes E.D. (1998). Amorphous Calcium Phosphate: Thermodynamic and Kinetic Considerations. Calcium Phosphates in Biological and Industrial Systems.

[7] Boskey A.L., Posner A.S. (1973). Conversion of amorphous calcium phosphate to microcrystalline hydroxyapatite. A pH-dependent, solution-mediated, solid-solid conversion. J. Phys. Chem..

[8] Meyer J.L., Weatherall C.C. (1982). Amorphous to crystalline calcium phosphate phase transformation at elevated pH. J. Colloid Interface Sci..

[9] Blumenthal N.C., Betts F., Posner A.S. (1977). Stabilization of amorphous calcium phosphate by Mg and ATP. Calcif. Tissue Res..

[10] Mekmene O., Quillard S., Rouillon T., Bouler J.-M., Piot M., Gaucheron F. (2009). Effects of pH and Ca/P molar ratio on the quantity and crystalline structure of calcium phosphates obtained from aqueous solutions. Dairy Sci. Technol..

[11] Zyman Z.Z., Rokhmistrov D., Glushko V.I. (2010). Structural and compositional features of amorphous calcium phosphate at the early stage of precipitation. J. Mater. Sci. Mater. Electron..

[12] Medvecky L., Sopcak T., Girman V., Briancin J. (2013). Amorphous calcium phosphates synthesized by precipitation from calcium D-gluconate solutions. Colloids Surfaces A Physicochem. Eng. Asp..

[13] Vecstaudza J., Locs J. (2017). Novel preparation route of stable amorphous calcium phosphate nanoparticles with high specific surface area. J. Alloys Compd..

[14] Vecstaudza J., Gasik M., Locs J. (2019). Amorphous calcium phosphate materials: Formation, structure and thermal behaviour. J. Eur. Ceram. Soc..

[15] Granados-Correa F., Bonifacio-Martínez J., Serrano-Gómez J. (2010). Synthesis and Characterization of Calcium Phosphate and Its Relation to Cr(VI) Adsorption Properties. Rev. Int. Contam. Ambient..

[16] Padmanabhan S.K., Balakrishnan A., Chu M.-C., Lee Y.J., Kim T.N., Cho S.-J. (2009). Sol–gel synthesis and characterization of hydroxyapatite nanorods. Particuology.

[17] Szatkowski T., Kołodziejczak-Radzimska A., Zdarta J., Szwarc-Rzepka K., Paukszta D., Wysokowski M., Ehrlich H., Jesionowski T. (2015). Synthesis and characterization of hydroxyapatite/chitosan composites. Physicochem. Probl. Miner. Process..

[18] Dorozhkin S. (2010). Green chemical synthesis of calcium phosphate bioceramics. J. Appl. Biomater. Biomech..

[19] Sinusaite L., Grigoraviciute-Puroniene I., Popov A., Ishikawa K., Kareiva A., Zarkov A. (2019). Controllable synthesis of tricalcium phosphate (TCP) polymorphs by wet precipitation: Effect of washing procedure. Ceram. Int..

[20] Cheshmehzangi A., Dawodu A. (2019). The Review of Sustainable Development Goals (SDGs): People, Perspective and Planning. Sustainable Urban Development in the Age of Climate Change.

[21] Dentin I. (1990). A New Method of Treatment for Dentin Hypersensitivity Precipitation of Calcium Phosphate in Situ. Dent. Mater. J..

[22] Gutiérrez-Arenas D.A., Cuca-García M., Méndez-Rojas M.A., Pro-Martínez A., Becerril-Pérez C.M., Mendoza-Álvarez M.E., Ávila-Ramos F., Ramírez-Bribiesca J.E. (2021). Designing Calcium Phosphate Nanoparticles with the Co-Precipitation Technique to Improve Phosphorous Availability in Broiler Chicks. Animals.

[23] Arifuzzaman S.M., Rohani S. (2004). Experimental Study of Brushite Precipitation. J. Cryst. Growth.

[24] Lim H.N., Kassim A., Huang N.M. (2010). Preparation and Characterization of Calcium Phosphate Nanorods Using Reverse Microemulsion and Hydrothermal Processing Routes. Sains Malays..

[25] Hutchens S. (2004). TRACE: Tennessee Research and Creative Exchange. Synthesis and Initial Characterization of a Calcium-Deficient Hydroxyapatite-Bacterial Cellulose Composite. Master’s Thesis.

[26] Katz L. (1964). X-ray diffraction in crystals, imperfect crystals, and amorphous bodies (Guinier, A.). J. Chem. Educ..

[27] Drouet C. (2013). Apatite Formation: Why It May Not Work as Planned, and How to Conclusively Identify Apatite Compounds. BioMed Res. Int..

[28] Christoffersen J., Christoffersen M.R., Kibalczyc W., Andersen F.A. (1989). A Contribution to the Understanding of the Formation of Calcium Phosphates. J. Cryst. Growth.

[29] Zhang H., Yan Y., Wang Y., Li S. (2002). Morphology and Formation Mechanism of Hydroxyapatite Whiskers from Moderately Acid Solution 111. Mater. Res..

[30] Ko H.-S., Lee S., Jho J. (2021). Synthesis and Modification of Hydroxyapatite Nanofiber for Poly(lactic acid) Composites with Enhanced Mechanical Strength and Bioactivity. Nanomaterials.

[31] Sugiura Y., Ishikawa K. (2018). Effect of Calcium and Phosphate on Compositional Conversion from Dicalcium Hydrogen Phosphate Dihydrate Blocks to Octacalcium Phosphate Blocks. Crystals.

[32] Sugiura Y., Makita Y. (2018). Sodium Induces Octacalcium Phosphate Formation and Enhances Its Layer Structure by Affecting the Hydrous Layer Phosphate. Cryst. Growth Des..

[33] Cheng F., Cao Q., Guan Y., Cheng H., Wang X., Miller J.D. (2013). FTIR analysis of water structure and its influence on the flotation of arcanite (K_2_SO_4_) and epsomite (MgSO_4_·7H_2_O). Int. J. Miner. Process..

[34] Hirsch A., Azuri I., Addadi L., Weiner S., Yang K., Curtarolo S., Kronik L. (2014). Infrared Absorption Spectrum of Brushite from First Principles. Chem. Mater..

[35] Coldea T.E., Socaciu C., Fetea F., Ranga F., Pop R.M., Florea M. (2013). Rapid Quantitative Analysis of Ethanol and Prediction of Methanol Content in Traditional Fruit Brandies from Romania, using FTIR Spectroscopy and Chemometrics. Not. Bot. Horti Agrobot..

[36] Van Der Post S.T., Hsieh C.-S., Okuno M., Nagata Y., Bakker H.J., Bonn M., Hunger J. (2015). Strong frequency dependence of vibrational relaxation in bulk and surface water reveals sub-picosecond structural heterogeneity. Nat. Commun..

[37] Burikov S., Dolenko T., Patsaeva S., Starokurov Y., Yuzhakov V. (2010). Raman and IR spectroscopy research on hydrogen bonding in water-ethanol systems. Mol. Phys..

[38] Xu J., Butler I.S., Gilson D.F. (1999). FT-Raman and high-pressure infrared spectroscopic studies of dicalcium phosphate dihydrate (CaHPO_4_·2H_2_O) and anhydrous dicalcium phosphate (CaHPO_4_). Spectrochim. Acta Part A Mol. Biomol. Spectrosc..

[39] Li C., Ge X., Li G., Bai J., Ding R. (2014). Crystallization of dicalcium phosphate dihydrate with presence of glutamic acid and arginine at 37 °C. Mater. Sci. Eng. C.

[40] Toshima T., Hamai R., Tafu M., Takemura Y., Fujita S., Chohji T., Tanda S., Li S., Qin G. (2014). Morphology control of brushite prepared by aqueous solution synthesis. J. Asian Ceram. Soc..

[41] Rubini K., Boanini E., Bigi A. (2019). Role of Aspartic and Polyaspartic Acid on the Synthesis and Hydrolysis of Brushite. J. Funct. Biomater..

[42] Koletzko B., Baker S., Cleghorn G., Neto U.F., Gopalan S., Hernell O., Hock Q.S., Jirapinyo P., Lonnerdal B., Pencharz P. (2005). Global Standard for the Composition of Infant Formula: Recommendations of an ESPGHAN Coordinated International Expert Group. J. Pediatr. Gastroenterol. Nutr..

[43] Komlev V.S., Fadeeva I.V., Fomin A.S., Shvorneva L.I., Ferro D., Barinov S.M. (2010). Synthesis of octacalcium phosphate by precipitation from solution. Dokl. Chem..

[44] Ivanov V.K., Fedorov P.P., Baranchikov A.Y., Osiko V.V. (2014). Oriented attachment of particles: 100 years of investigations of non-classical crystal growth. Russ. Chem. Rev..

[45] van Santen R.A. (1984). The Ostwald Step Rule. J. Phys. Chem..

[46] Iijima M. (2020). Modification of octacalcium phosphate growth by enamel proteins, fluoride, and substrate materials and influence of morphology on the performance of octacalcium phosphate biomaterials. Octacalcium Phosphate Biomater..

[47] Toby B.H. (2006). R factors in Rietveld analysis: How good is good enough?. Powder Diffr..

[48] Para T.A., Sarkar S.K. (2021). Challenges in Rietveld Refinement and Structure Visualization in Ceramics. Adv. Ceram. Mater..

