# Supplementary material for: The Influence of Residuals Combining Temperature and Reaction Time on Calcium Phosphate Transformation in a Precipitation Process
Source: J Funct Biomater. 2022 Jan 19;13(1):9. doi: 10.3390/jfb13010009 (PMC8883985; doi:10.3390/jfb13010009)
Supplement: Supplementary file 1 [file jfb-13-00009-s001.zip › jfb-1494170-SI.pdf]

Figures below are SEM images of samples made via different synthesis conditions as explained in table 1, that are added to provide complementary information.

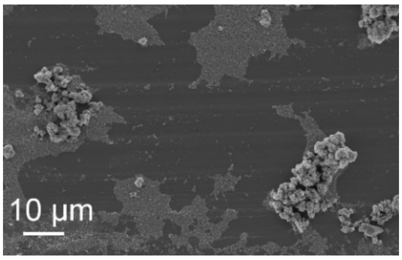

Figure S1. SEM images of the sample made at 21°C/W/1.

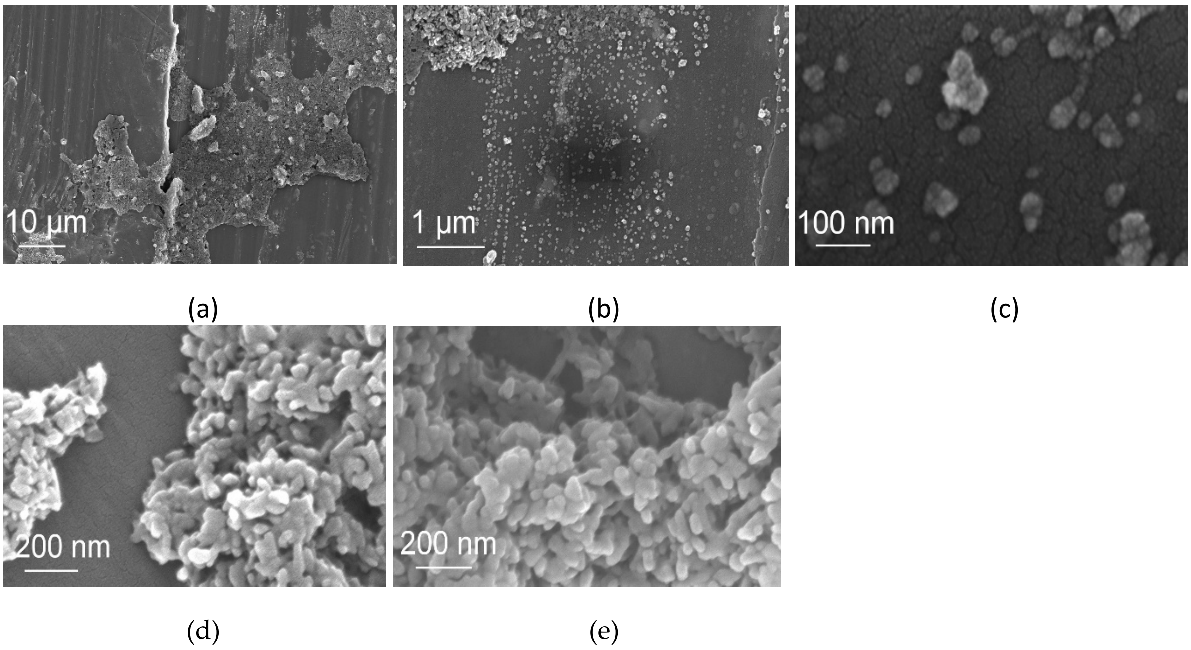

Figure S2. (a-e) SEM images of the sample made at 21°C/W/3.

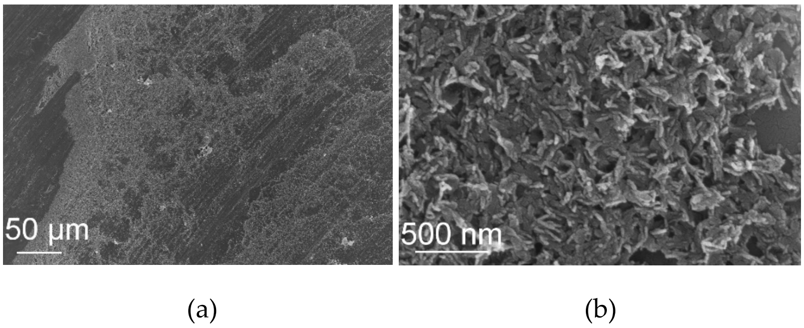

Figure S3. SEM images of the sample made at (a) 21°C/W/30 and (b) 21°C/W/60.

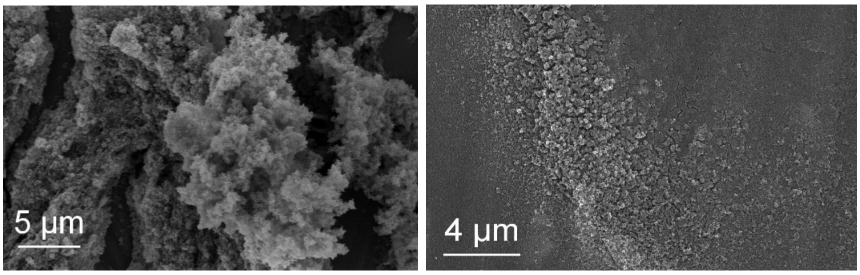

(a) (b)

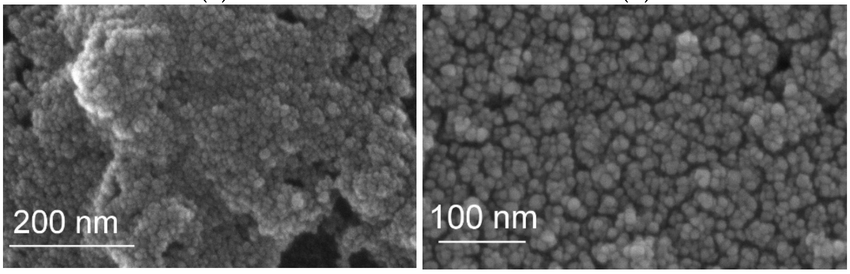

(c) (d)

Figure S4. (a-d) SEM images of samples made at 21°C/NotW/1.

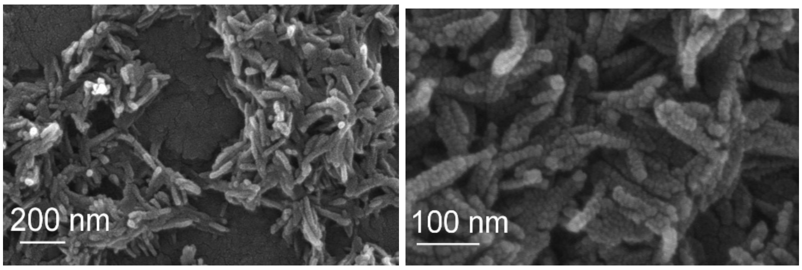

(a) (b)

Figure S5. SEM images of (a-b) samples made at 60°C/NotW/1.

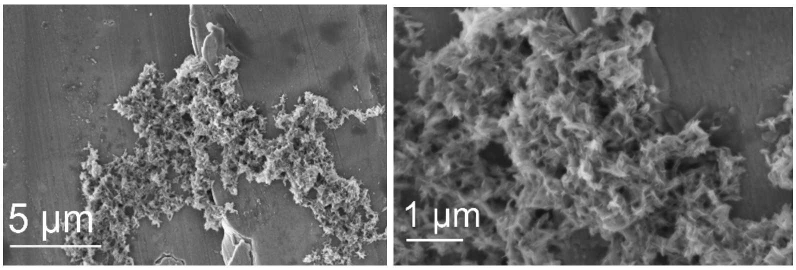

(a) (b)

Figure S6. SEM images of (a-b) samples made at 60°C/NotW/3.

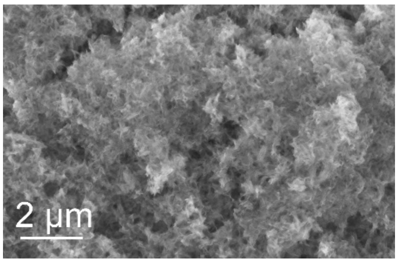

Figure S7. SEM images of (a-b) samples made at 60°C/NotW/30

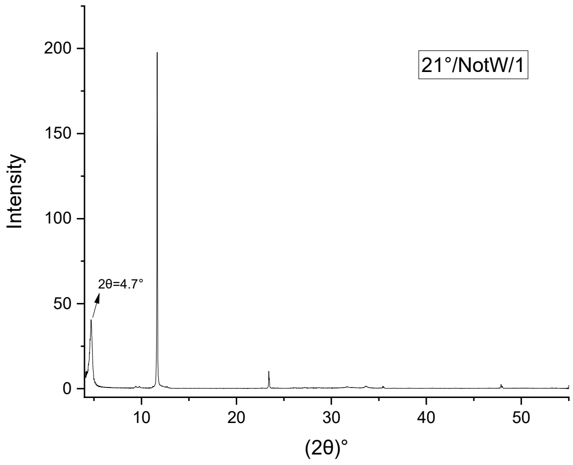

Figure S8 XRD analysis conducted to observe the existence peaks in low angels of 2θ.
